# Complete Chloroplast Genome of Topotype Material of the Coast Live Oak *Quercus agrifolia* Née var. *agrifolia* (Fagaceae) from California

**DOI:** 10.1128/mra.00004-22

**Published:** 2022-03-07

**Authors:** Adam N. Garcia, Jennifer Hernandez Ramos, Aileen G. Mendoza, Asmahan Muhrram, Jessica M. Vidauri, Jeffery R. Hughey

**Affiliations:** a Division of Mathematics, Science, and Engineering, Hartnell College, Salinas, California, USA; University of California, Riverside

## Abstract

Here, we present the chloroplast genome sequence of Quercus agrifolia Née, the California live oak, an ecologically important oak species along the coast of California. The genome is 161,283 bp in length, encodes 132 genes, and has a high level of gene synteny to other Fagaceae.

## ANNOUNCEMENT

Quercus agrifolia Née, the California live oak or coast live oak, is an evergreen described originally by Luis Née from Monterey, CA (1, 2). The species was said to have sessile axillary fruits and glabrous leaves that were broad, ovate, subcordate, and toothed (1). *Quercus agrifolia* is distributed from northern California to Baja California, Mexico, where it occurs in valleys and slopes in mixed-evergreen forests and woodlands at elevations less than 1,440 meters (3). In addition to the autonym *Q. agrifolia* var. *agrifolia*, two varieties have been proposed, namely, *Q. agrifolia* var. *oxyadenia* (Torr.) J.T. Howell and *Q. agrifolia* var. *frutescens* Engelm. *Q. agrifolia* var. *oxyadenia* is accepted currently while *Q. agrifolia* var. *frutescens* is considered a synonym of *Q. agrifolia* var. *agrifolia* (3). Based on genetic analyses, *Q. agrifolia* is closely related to Quercus parvula Greene and Quercus wislizeni A. DC. (4–6). More than 30 oak chloroplast genomes have been sequenced (7–9); however, the *Q. agrifolia* genome has not been deciphered. In this study, we assembled and characterized the complete chloroplast genome sequence of *Q. agrifolia* var. *agrifolia* to contribute to the bioinformatics and systematics of this variety and subsection Agrifoliae.

The topotype specimen of *Q. agrifolia* var. *agrifolia* analyzed here was collected from the Presidio in Monterey, CA (36°36′21.9″N 121°53′51.9″W), and deposited at Hartnell College under voucher number HCC 267. The DNA was extracted using the DNeasy blood and tissue kit (Qiagen) following several modifications (10). The binding step during centrifugation was reduced to 4,000 × *g* for 3 minutes, and the DNA was eluted after incubation for 7 minutes in 40 μL Tris-acetate-EDTA (TAE). The 150-bp paired-end PE library was constructed with the NEBNext Ultra II DNA library prep kit (New England BioLabs) and sequenced by Novogene on an Illumina NovaSeq 6000 instrument. The analysis yielded 56,418,120 reads. The genome sequence was assembled by mapping the raw reads onto the reference genome Quercus coccinea, GenBank accession number MN308055 (11), using the medium-low sensitivity/fast setting in Geneious Prime 2019.1.3 (Biomatters Limited) resulting in an average coverage of 4,323×. The gaps were closed by iterative mapping using the same settings in Geneious Prime. The annotation was completed using the default settings in GeSeq (12), followed by manual adjustments according to NCBI ORFfinder and Sequin 15.5 (13). Pairwise distances were calculated using the default settings in DIVEIN (14).

**FIG 1:**
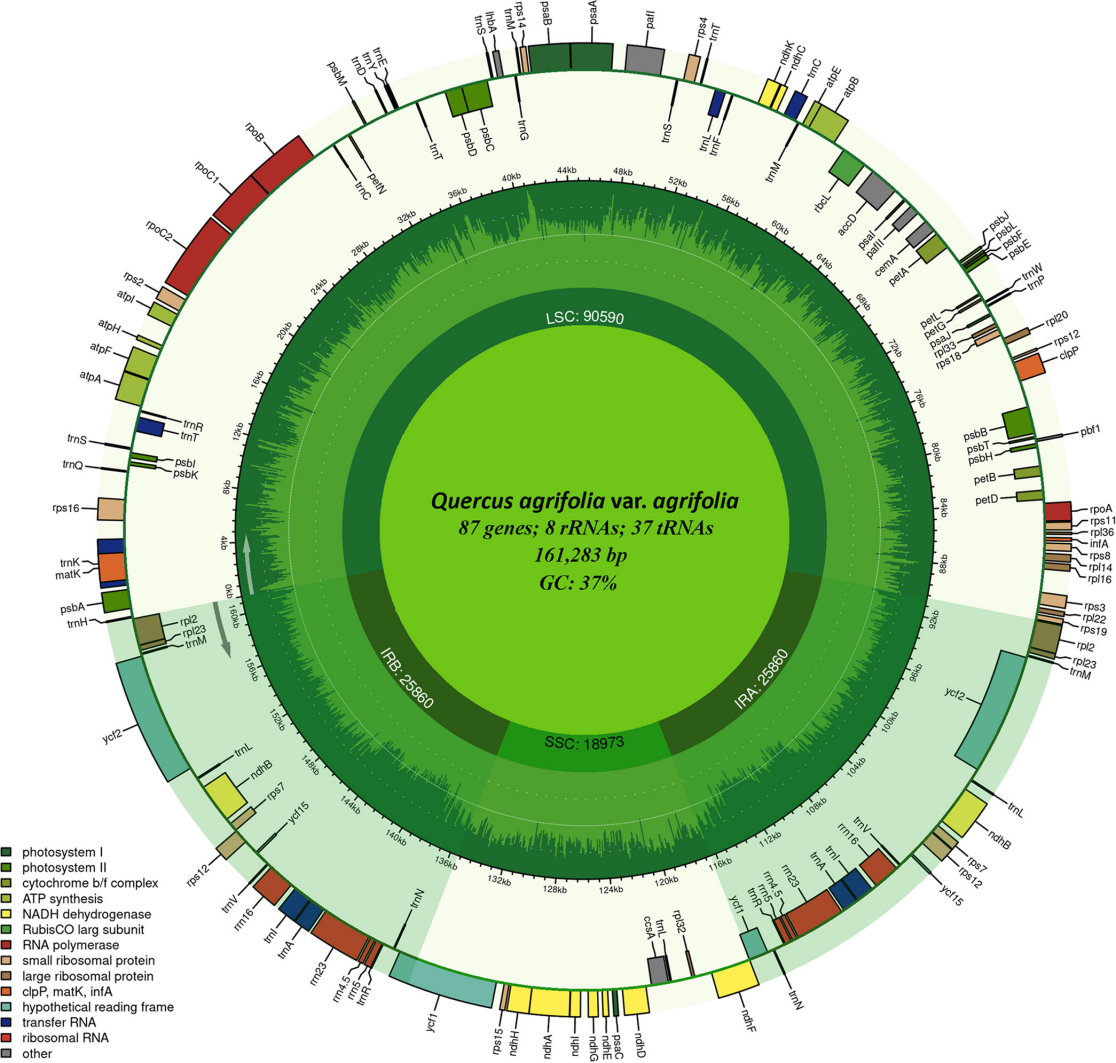
Complete chloroplast genome of *Quercus agrifolia* var. *agrifolia*. The genome was annotated using GeSeq (12), NCBI ORFfinder, and Sequin 15.5 (13) and mapped with Chloroplot (16). The innermost ring identifies the LSC, SSC, and the two inverted repeats. The next ring displays the GC content and direction of transcription, as indicated by the two arrows. The final ring shows the genes. Genes transcribed clockwise are on the inside, while counterclockwise transcriptions are on the outside. The color coding corresponds to genes of different groups as listed in the key in the bottom left.

The complete chloroplast genome of *Q. agrifolia* var. *agrifolia* is 161,283 bp in length and displays the characteristic flowering plant quadripartite structure (15). The genome contains a large single-copy region (LSC), small single-copy region (SSC), and two inverted repeats (IRs) with lengths of 90,590 bp, 18,973 bp, and 25,860 bp, respectively (Fig. 1). The GC content is 37.0%. The genome contains 132 genes, including 87 protein-coding, 37 tRNA, and 8 rRNA genes. Thirteen of the genes contain one intron (*atpF*, *clpP*, *ndhA*, *ndhB*, *rpl2*, *rpoC1*, *rps16*, *trnA*, *trnC*, *trnE*, *trnK*, *trnL*, and *trnT*) and two genes contain two introns (*pafI* and *rps12*). Gene content and organization are identical to other oaks classified in section Lobatae (7, 9, 11). A comparison of the chloroplast genome of *Q. agrifolia* var. *agrifolia* to other Lobatae found it to be 99.79% similar in nucleotide sequence to *Q. rubra*, 99.78% to *Q. palustris*, and 99.77% to *Q. coccinea*.

This chloroplast genome will be useful for examining species-level relationships and *Quercus* systematics, as well as helping to resolve longstanding hypotheses regarding oak hybridization in Lobatae.

### Data availability.

The complete chloroplast genome sequence of *Quercus agrifolia* var. *agrifolia* is available in GenBank under accession number OK634019. The associated BioProject, SRA, and BioSample numbers are PRJNA773816, SRX12744819, and SAMN22513939, respectively. The reference genome for the annotation was *Q. coccinea* (GenBank accession number MN308055).
